# Supplementary material for: Sequence and structural variations determining the recruitment of WNK kinases to the KLHL3 E3 ligase
Source: Biochem J. 2022 Mar 4;479(5):661–75. doi: 10.1042/BCJ20220019 (PMC9022995; doi:10.1042/BCJ20220019)
Supplement: Supplementary Figure S1-S7 [file BCJ-479-661-s1.pdf]

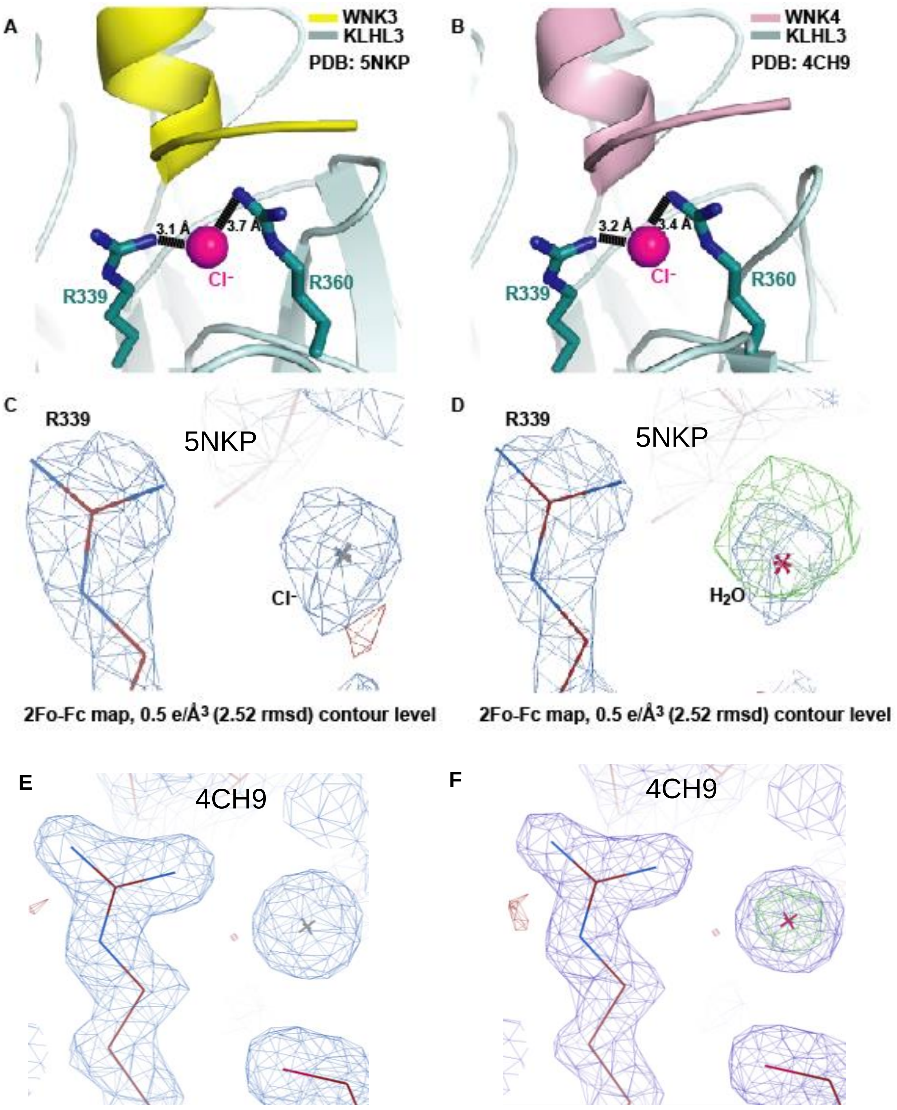

Figure S1. Bound chloride ions in the KLHL3-WNK3 and KLHL3-WNK4 structures. (**A** and **B**) A bound chloride ion is observed in the KLHL3-WNK3 and KLHL3-WNK4 complexes and forms electrostatic interactions with positive charged Arg339 and Arg360 in KLHL3. KLHL3 residues are labelled in green, WNK3 peptide is shown in yellow, and WNK4 peptide is shown in pale pink. The chloride ions are shown as magenta spheres. The electrostatic interactions are demonstrated using dashed lines. The distances (Å) are indicated. (**C-F**) Stick representation and 2Fo-Fc electron density maps contoured at 0.5 σ (2.52 rmsd) for KLHL3 Arg339 and the interfacing Cl^- (**C and E**) or a replaced water molecule (**D** and **F**) in the indicated structures. Blue meshes represent the experimental electron density. The green mesh around the water molecule represents a positive discrepancy comparing experimental data with the structure model.

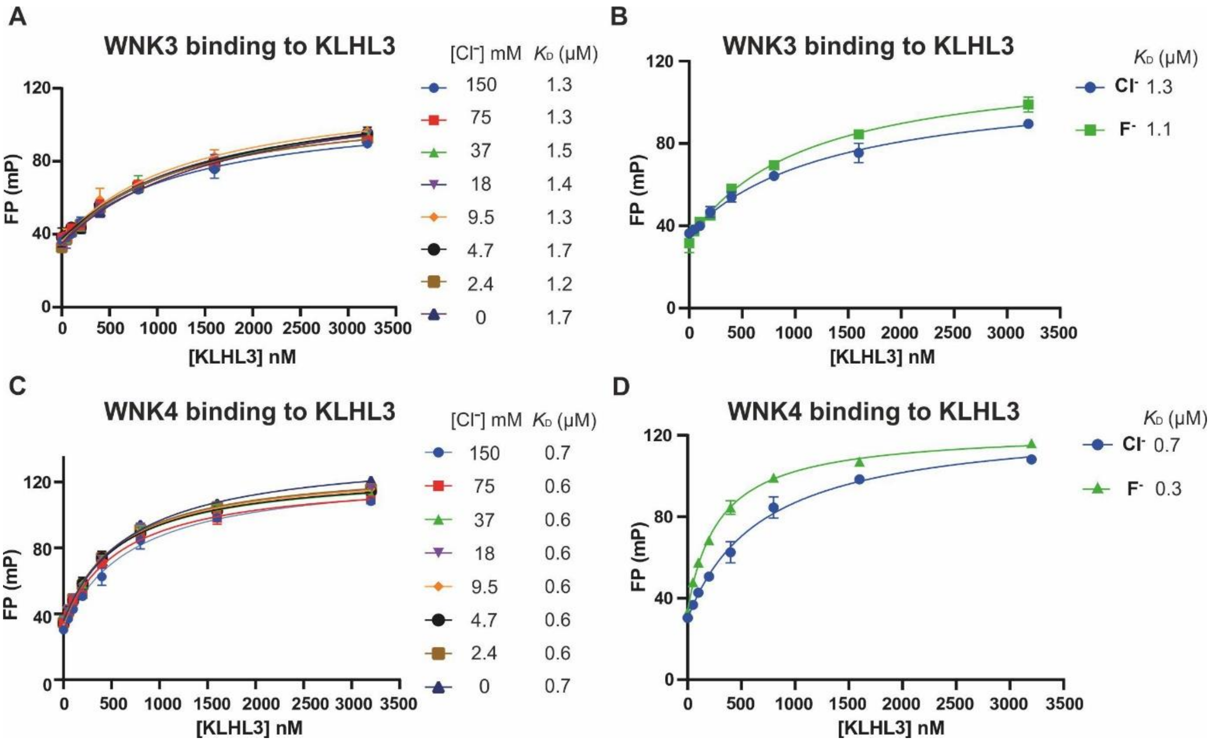

Figure S2. The effect of chloride on the interaction between KLHL3 and WNK peptides. (**A-D**) Analysis of the interaction between KLHL3 and WNK peptides by fluorescence polarisation in buffers containing various Cl^- concentrations. Purified KLHL3 (a.a. 298-587) was diluted appropriately and mixed at a 1:1 volume ratio with 20 nM Rhodamine-110 fluorophore labelled WNK3 or WNK4 peptides to the concentration stated in the Figure, with the peptide concentration consistent at 10 nM. In panels **A** and **C**, buffers containing various Cl^- concentrations were balanced with phosphate buffer to keep a constant ionic strength of 154 mM. In panels **B** and **D**, 150 mM Cl^- or 150 mM F^- buffers were tested. Fluorescence polarisation measurements were recorded and corrected to the fluorescent probe alone. Each data point represents two technical replicates. For data analysis, one site-total with constant NS (slope of non-specific binding) equal to zero was assumed (model $Y=Bmax*X/(K_D+X)$ + Background) and the disassociation constant was obtained. Binding curves were then generated with Prism6 using milli-polarization (mP) units.

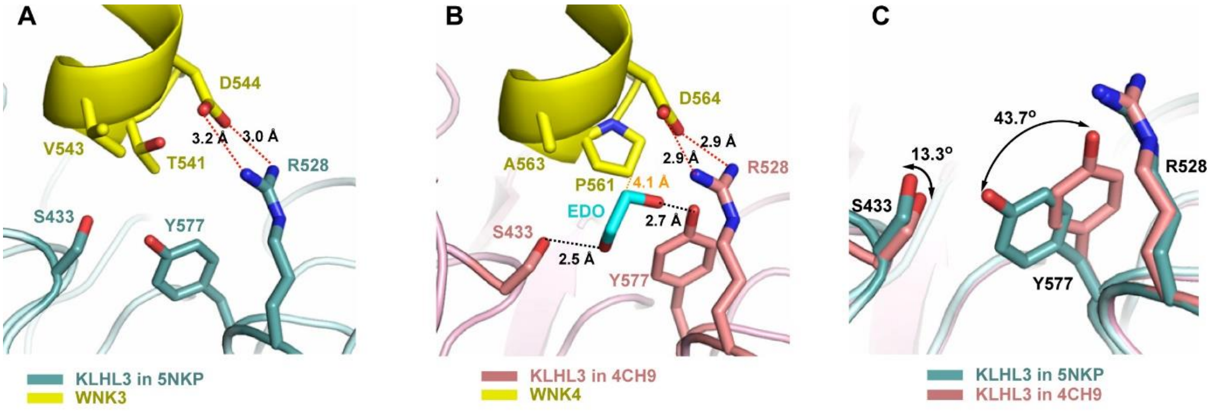

Figure S3. Side chain packing differences around the key salt bridge between WNKs and KLHL3 Arg528. (**A**) KLHL3 Arg528 forms a key salt bridge interaction with WNK3 Asp544. Selected side chains in the vicinity are displayed in stick representation, including KLHL3 Tyr577 as well as WNK3 Thr541 and Val543. KLHL3 is shown in green. WNK3 is shown in yellow. Salt bridge interactions are indicated with red dashed lines and labelled with distance measurements. (**B**) In the equivalent WNK4 complex, KLHL3 Arg528 forms a salt bridge with WNK4 Asp564. The diverged WNK4 degron residues Pro561 and Ala563 allowed an ethylene glycol (EDO) molecule from the cryo-protectant to pack in the crystal lattice adjacent to the salt bridge and to form hydrogen bonds with the side chains of KLHL3 Ser433 and Tyr577, as indicated with black dashed lines and distance measurements. The distance between WNK4 Pro561 and the EDO is also indicated in orange. KLHL3 is shown in red, WNK4 in yellow. (**C**) Superposition of the two KLHL3-WNK complexes highlighting the KLHL3 residues Ser433, Arg528 and Tyr577. Side chain packing in the vicinity of KLHL3 Arg528 is altered by the WNK3-specific substitutions of Thr541 and Val543 and the shift in the position of KLHL3 Tyr577. KLHL3 in PDB 5NKP (KLHL3-WNK3 co-structure) is coloured in green and KLHL3 in PDB 4CH9 (KLHL3-WNK4 co-structure) is coloured in red.

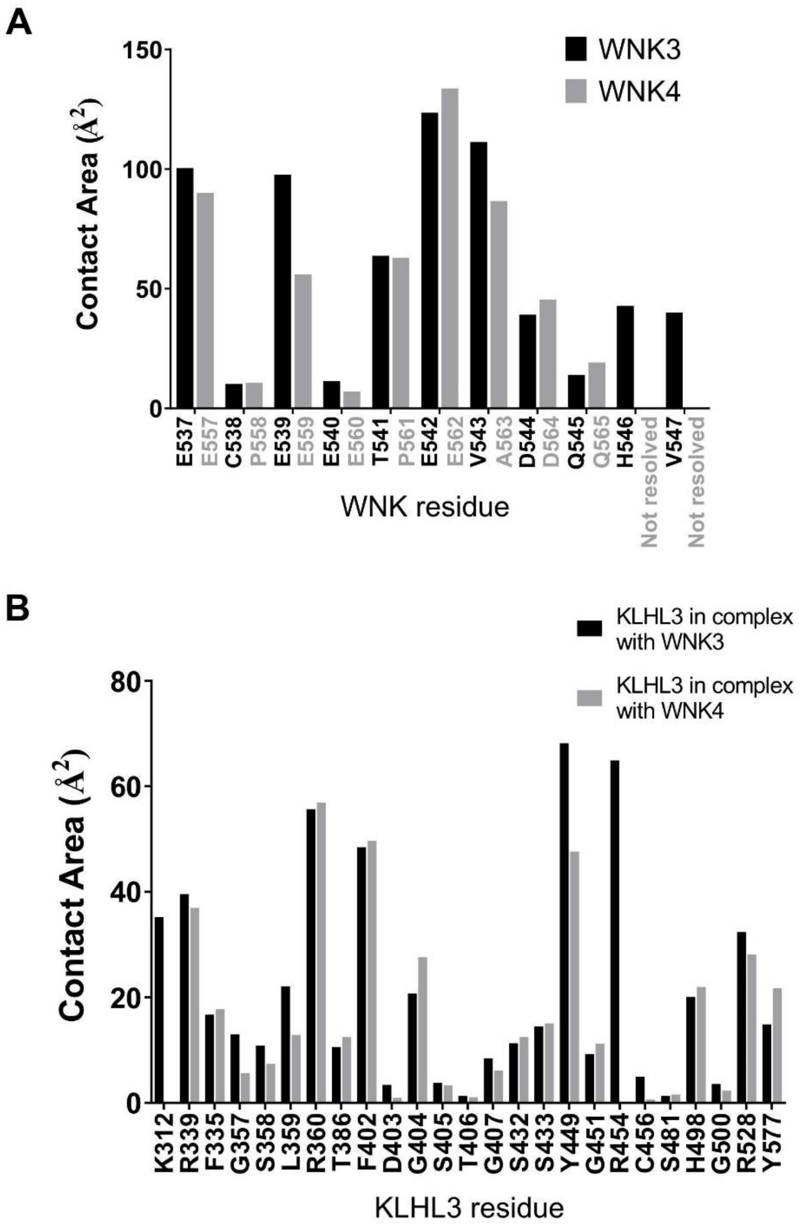

Figure S4. Comparison of contact residues in the KLHL3-WNK3 and KLHL3-WNK4 complex structures. (**A**) Buried interface surface areas for interacting residues in the WNK3 (black bars) and WNK4 (grey bars) peptide sequences, respectively. The side chain of Glu559 was only partially resolved in the KLHL3-WNK4 structure. (**B**) Buried interface surface areas for interacting residues in KLHL3 in the WNK3 (black bars) and WNK4 (grey bars) complex structures, respectively. For KLHL3 Lys312, the interacting residues in WNK4 were not fully resolved.

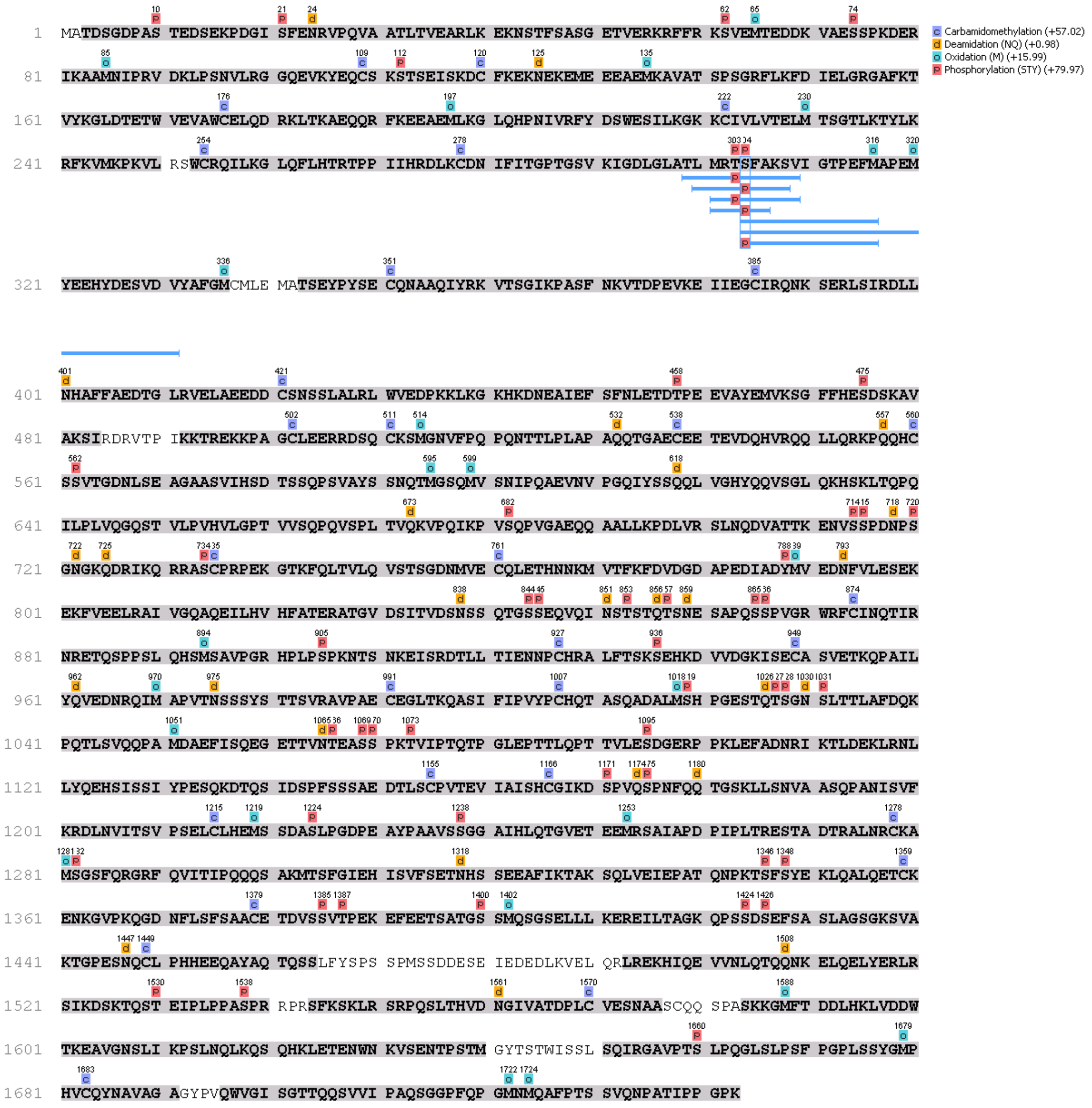

Figure S5. Overview of WNK3 peptide hits from hypotonic treatment. Residues recovered in elastase MS/MS are shaded in grey. Modifications are labelled above residues. Peptide hits in the kinase activation segment are illustrated.

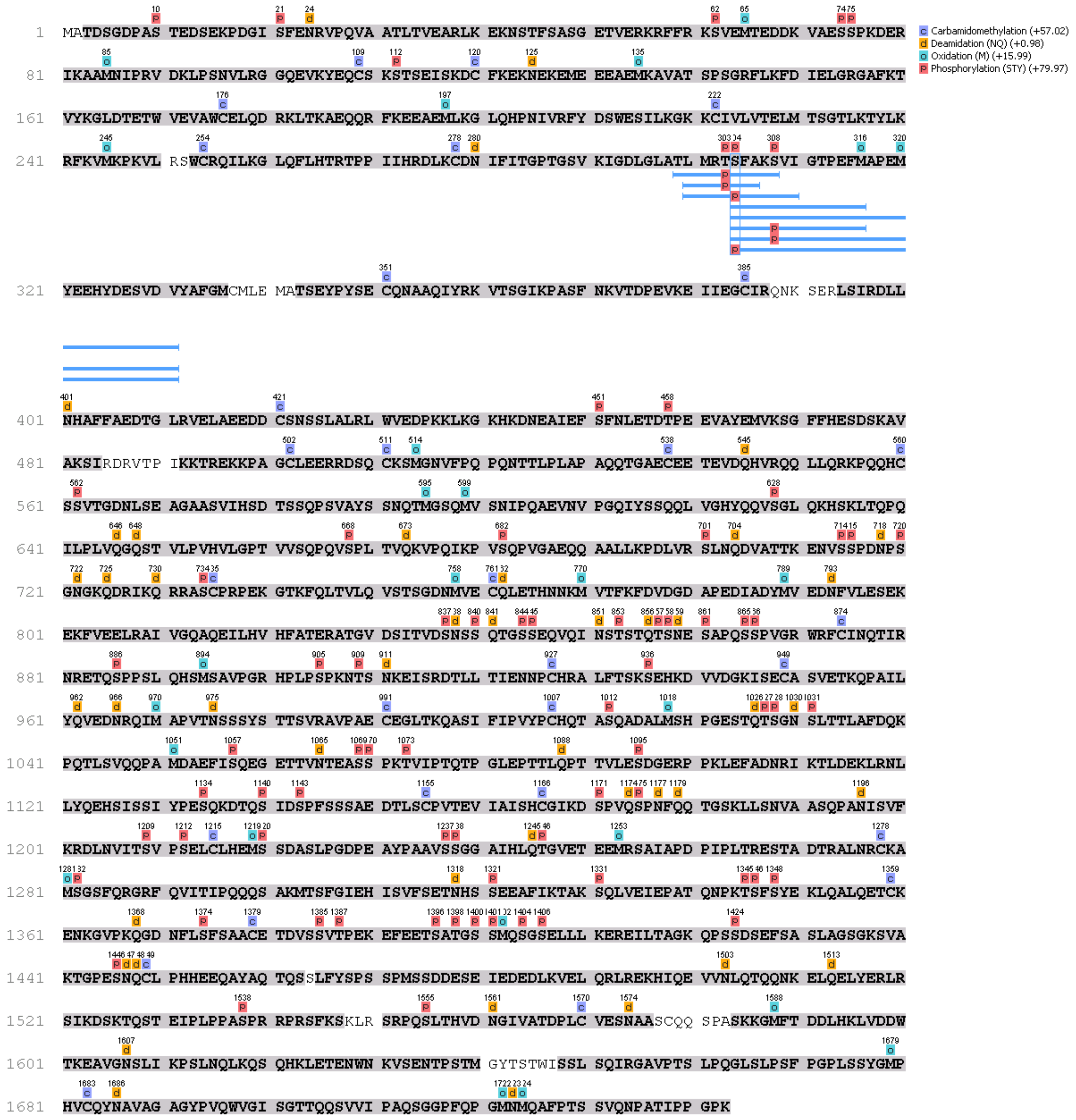

Figure S6. Overview of WNK3 peptide hits from isotonic treatment. Residues recovered in elastase MS/MS are shaded in grey. Modifications are labelled above residues. Peptide hits in the kinase activation segment are illustrated.

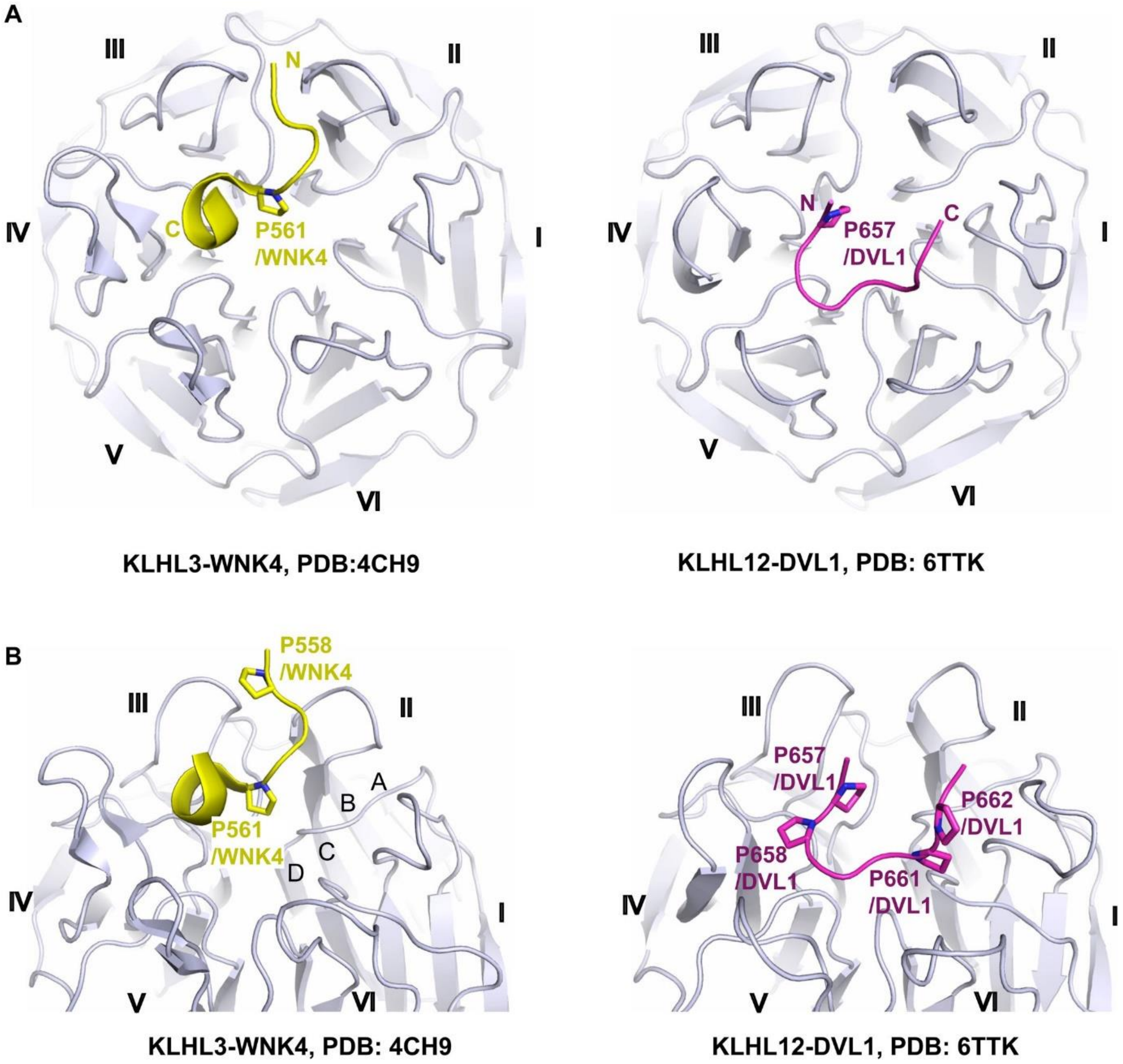

Figure S7. Diverse binding modes of Kelch-interacting PXXP motifs in substrate degrons. (**A**) Overviews of the KLHL3-WNK4 and KLHL12-DVL1 co-structures showing the substrate peptide conformations that contact multiple blades in the Kelch domains. WNK4 Pro561 and DVL1 Pro657 in stick representation both anchor the degron peptides in the centre of the Kelch domain binding pockets. Kelch domains are shown in grey, WNK4 in yellow and DVL1 in purple. N and C-termini of the degron peptides are labelled. Blades I to VI of the β-propeller are labelled for each Kelch domain. (**B**) The proline-rich motifs in the peptide degrons bind to distinct parts of the Kelch domains. Prolines in each degron motif are shown in stick representation. Blades I to VI and the β-strands A to D in Blade II are labelled for the Kelch domain.
